# Associations between school sports atmosphere and college students’ mental well-being: the chain mediation role of growth mindset and psychological resilience

**DOI:** 10.3389/fpsyg.2026.1941712

**Published:** 2026-10-07

**Authors:** Yue Liu

**Affiliations:** Heze Medical College, Heze, Shandong, China

**Keywords:** college students’ mental well-being, growth mindset, psychological resilience, school sports atmosphere, structural equation modeling

## Abstract

**Background:**

College students’ mental health is affected by academic pressure, employment competition, interpersonal adjustment, and uncertainty about the future. Previous research has focused mainly on exercise frequency, intensity, and participation, with less attention to school sports atmosphere as a contextual campus factor. Based on the social ecological model, cognitive appraisal theory, and the stress-buffering model, this study examined the relationship between school sports atmosphere and college students’ mental well-being and tested the direct association and indirect effects involving growth mindset and psychological resilience.

**Methods:**

A questionnaire survey was conducted among 635 Chinese college students. Participants completed the School Sports Atmosphere Scale, Growth Mindset Scale, Chinese Connor–Davidson Resilience Scale, and Chinese WHO-5 Well-Being Index. Descriptive statistics were conducted in SPSS 28.0, and structural equation modeling was performed in AMOS 28.0 to test the theory-specified structural associations and indirect effects.

**Results:**

School sports atmosphere was positively associated with mental well-being, growth mindset, and psychological resilience. Growth mindset was positively associated with psychological resilience, and psychological resilience was positively associated with mental well-being. However, the direct path from growth mindset to mental well-being was not significant.

**Conclusion:**

A positive school sports atmosphere was directly associated with better mental well-being and indirectly associated with mental well-being through psychological resilience. Although growth mindset did not show a significant independent indirect effect, it contributed to a significant sequential indirect effect through psychological resilience. Growth mindset was associated with mental well-being primarily through its association with psychological resilience rather than through a significant direct pathway. Universities should integrate improvements in the school sports environment with interventions that cultivate growth mindset and psychological resilience.

## Introduction

College students’ mental health has attracted considerable attention in the fields of psychology and higher education. Owing to multiple challenges, including academic pressure, employment competition, interpersonal adjustment, and changes in living environments, college students may experience persistently high levels of psychological strain, which can increase their risk of mental health problems such as anxiety and depression (Sheldon et al., 2021). Mental health refers to a comprehensive state in which individuals maintain adaptive cognitive, emotional, and behavioral functioning, effectively cope with everyday stress, realize their potential, and sustain positive psychological functioning (Coronel-Santos and Rodríguez-Macías, 2022). College students’ mental health not only affects their academic engagement, quality of life, and social adjustment but may also have profound implications for their future career development. In the present study, however, the focal outcome is positive mental well-being as operationalized by the WHO-5, rather than the broader construct of mental health or psychopathology.

College students’ mental health is generally shaped by the combined influence of multiple factors, including individual psychological characteristics, family and social support, the school environment, and lifestyle behaviors (Campbell et al., 2022). In recent years, the relationship between physical activity and mental health has received increasing scholarly attention. However, previous studies have predominantly focused on behavioral factors, such as the frequency of sports participation, exercise intensity, and exercise habits, while paying insufficient attention to school sports atmosphere as a persistent and contextual feature of the campus environment (Huang et al., 2024). School sports atmosphere reflects not only external conditions, such as sports facilities, physical education curricula, and opportunities for participation in sports activities, but also the psychosocial environment, including support from physical education teachers, peer interactions, fairness of rules, and sports culture (Wu et al., 2024). As such, a positive school sports atmosphere may provide students with environmental and interpersonal resources that are associated with better mental well-being. Examining this relationship therefore has both theoretical and practical significance.

### Research gap

Recent research has begun to examine school sports atmosphere directly. For example, Li et al. (2026) investigated the relationship between school sports atmosphere and social anxiety among Chinese college students, identifying exercise self-efficacy as a mediator and gender as a moderator (Li et al., 2026). This work demonstrates the relevance of school sports atmosphere to students’ psychological outcomes, but important questions remain. First, previous research has focused mainly on negative psychological outcomes, such as social anxiety, whereas less is known about its relationship with positive mental well-being. Second, the psychological processes linking school sports atmosphere with mental well-being remain insufficiently understood. In particular, the combined roles of growth mindset and psychological resilience have rarely been examined within the same explanatory model. The present study therefore extends previous research by examining a distinct positive psychological outcome and by testing whether growth mindset and psychological resilience form specific and sequential indirect pathways between school sports atmosphere and college students’ mental well-being.

### Theoretical framework

The proposed model draws on complementary theoretical perspectives. The Social Ecological Model provides a basis for considering school sports atmosphere as an environmental context associated with students’ psychological functioning. Cognitive Appraisal Theory provides a basis for understanding growth mindset as a cognitive orientation toward challenge and change, whereas the Stress-buffering Model provides a basis for understanding psychological resilience as an adaptive resource under stress. Together, these perspectives provide the theoretical foundation for examining the proposed sequence among school sports atmosphere, growth mindset, psychological resilience, and mental well-being.

### Literature review by variables

#### The relationship between school sports atmosphere and college students’ mental well-being

School sports atmosphere refers to the overall contextual characteristics and psychological experiences related to sports activities that individuals perceive within a specific environment (Zheng et al., 2023). Such contextual experiences may arise from external environmental factors, including campus sports facilities, organizational support, and peer participation, as well as from individuals’ subjective perceptions, such as participation motivation and emotional experiences. A positive school sports atmosphere is generally characterized by supportive interpersonal interactions, a favorable social environment, and active participation, thereby providing individuals with motivation to engage in sports activities (Wu et al., 2024).

According to the Social Ecological Model, individual behavior and psychological states are jointly influenced by environmental factors at multiple levels, among which contextual environments play an important role in shaping health-related outcomes (Kousoulis and Goldie, 2021). Previous studies have shown that when individuals are situated in a positive and supportive sports environment, their subjective well-being and emotional stability are likely to improve (Lochbaum and Sisneros, 2024). Therefore, school sports atmosphere may be associated with mental well-being through enhancing individuals’ emotional experiences and the quality of their social interactions.

From the perspective of Self-Determination Theory, individuals are more likely to develop intrinsic motivation and maintain positive psychological functioning in environments that support autonomy, competence, and relatedness (Grassinger et al., 2024). For college students, continued exposure to a positive school sports atmosphere may enhance their psychological need satisfaction and sense of belonging, thereby reducing the risk of mental health problems. Accordingly, the following hypothesis is proposed:

*H1*: School sports atmosphere is positively associated with college students’ mental well-being.

#### The indirect role of growth mindset

Some researchers have argued that individuals’ mental health is not determined entirely by external environmental conditions but results from the interaction between environmental factors and individual cognition (Riepenhausen et al., 2022). Even when individuals are exposed to the same situation, they may not necessarily experience the same psychological outcomes. Therefore, an additional variable should be considered to provide a more comprehensive explanation of the potential pathway linking school sports atmosphere with college students’ mental well-being. Cognitive Appraisal Theory proposes that individuals’ subjective appraisals of environmental stimuli play a central role in the relationship between stress and psychological outcomes (Gomes et al., 2022). According to this theory, when college students encounter academic and daily-life stressors, maladaptive appraisals may lead to negative emotions and adversely affect their mental health.

Growth mindset refers to the belief that one’s abilities can be continuously developed and improved through effort and learning, and is commonly described as a positive and dynamic view of ability (Zhang et al., 2024). Research has shown that individuals with a stronger growth mindset demonstrate greater adaptability when encountering difficulties and setbacks (Zhang and Li, 2025). A positive school sports atmosphere may encourage college students to perceive their abilities as malleable and improvable, thereby facilitating the development of a growth mindset. Although evidence for a direct association between growth mindset and college students’ mental well-being remains limited, previous studies have indicated that growth mindset may help strengthen psychological resilience and self-efficacy, thereby reducing the adverse effects of stress (Cherewick et al., 2023). It is therefore reasonable to propose a specific indirect effect of school sports atmosphere on college students’ mental well-being through growth mindset. Accordingly, the following hypothesis is proposed:

*H2*: There is a positive specific indirect effect of school sports atmosphere on college students’ mental well-being through growth mindset.

#### The indirect role of psychological resilience

Psychological resilience refers to an individual’s capacity to adapt effectively and maintain positive psychological functioning when confronted with stress and adversity. It reflects the ability to regulate emotions and behaviors adaptively in complex or challenging environments (Masten et al., 2021). Structurally, psychological resilience is generally considered a multidimensional construct that encompasses characteristics such as tenacity, adaptability, optimism, and self-regulation (de la Fuente et al., 2022). Psychological resilience is therefore important for explaining how individuals cope with stress and maintain their mental health.

According to the Stress-buffering Model, psychological resilience can mitigate the adverse effects of external stressors on mental health (Liu et al., 2021). This model emphasizes that individuals respond to external stress based partly on the psychological resources available to them. When confronted with academic pressure, interpersonal conflict, or uncertainty about the future, college students with insufficient psychological resilience may experience difficulty regulating their emotions effectively, thereby increasing their risk of psychological problems such as anxiety and depression (Mastrokoukou et al., 2024). In contrast, higher levels of psychological resilience may help individuals maintain emotional stability and psychological balance under stressful circumstances.

Within the school environment, a positive school sports atmosphere may provide students with opportunities to regulate their emotions and alleviate stress. Participation in sports activities may enable students to express and manage their emotions, thereby contributing to the development of psychological resilience (Liu et al., 2025). In particular, a supportive school sports atmosphere characterized by high levels of participation may help students develop more adaptive strategies for coping with stress and strengthen their psychological adjustment (Zhang and Li, 2025). Therefore, psychological resilience may represent an important indirect pathway linking school sports atmosphere with college students’ mental well-being. Accordingly, the following hypothesis is proposed:

*H3*: There is a positive specific indirect effect of school sports atmosphere on college students’ mental well-being through psychological resilience.

#### The chain mediation effect of growth mindset and psychological resilience

In research on the relationship between school sports atmosphere and college students’ mental well-being, considering growth mindset and psychological resilience together in a theoretically specified sequence may provide a more comprehensive explanation of how the external sports environment is associated with mental well-being through individuals’ internal psychological resources. School sports atmosphere represents not only the external conditions that support college students’ participation in sports activities but also a sustained contextual resource within the campus environment, as reflected in support from physical education teachers, peer interactions, and opportunities for sports participation (Guo et al., 2023; Rocliffe et al., 2024). A positive school sports atmosphere may provide students with more opportunities to experience success and enable them to recognize the progress resulting from their efforts during sports participation (Wu et al., 2024).

Growth mindset may further contribute to the development of psychological resilience. When confronted with academic pressure or failure in sports competitions, college students with a growth mindset are more likely to regard difficulties as challenges that can be overcome (Yang X. J. et al., 2025). Such positive cognition may reduce feelings of helplessness and avoidance behaviors. As an important psychological resource, psychological resilience can help college students maintain psychological balance when facing stress and setbacks, alleviate the adverse effects of negative emotions on mental health, and promote positive psychological states and subjective well-being (Brett et al., 2023).

Unlike models that treat growth mindset and psychological resilience as two independent mediators, the proposed model specifies a sequential association between them. Growth mindset mainly reflects individuals’ cognitive beliefs about difficulties and failure, whereas psychological resilience reflects their ability to adapt and adjust in stressful situations (Zhang and Li, 2025). Based on theory, a positive school sports atmosphere may be associated with a stronger growth mindset, which may in turn be associated with greater psychological resilience and, subsequently, higher mental well-being. For example, in a highly supportive school sports environment, students may be more likely to interpret failures or difficulties encountered during sports activities as opportunities for growth (Zheng et al., 2023). Such a cognitive orientation may be associated with greater persistence and adaptive coping. Because all variables were measured concurrently, this ordering should be interpreted as theory-specified rather than as evidence of temporal causation.

Taken together, the Social Ecological Model, Cognitive Appraisal Theory, and Stress-buffering Model provide a coherent explanatory framework. The Social Ecological Model positions school sports atmosphere as a contextual campus resource; Cognitive Appraisal Theory explains how students’ interpretations and growth-oriented beliefs may be associated with that context; and the Stress-buffering Model positions psychological resilience as an adaptive resource associated with functioning under stress. The proposed model therefore tests whether these constructs are linked in the theoretically specified sequence while recognizing that the cross-sectional design cannot establish temporal order or causality. Based on the above analysis, the following hypothesis is proposed:

*H4*: There is a positive sequential indirect effect of school sports atmosphere on college students’ mental well-being through growth mindset and psychological resilience.

In summary, to better explain the relationship between school sports atmosphere and college students’ mental well-being, this study developed a theory-specified cross-sectional model, as shown in Figure 1. The study examined the direct association between school sports atmosphere and mental well-being and tested the specific and sequential indirect effects involving growth mindset and psychological resilience. The direct paths are interpreted as cross-sectional associations, and the indirect effects are treated as statistical estimates within the theory-specified model rather than evidence of a temporal causal chain. The findings may provide a useful reference for interventions aimed at supporting college students’ mental well-being.

**Figure 1:**
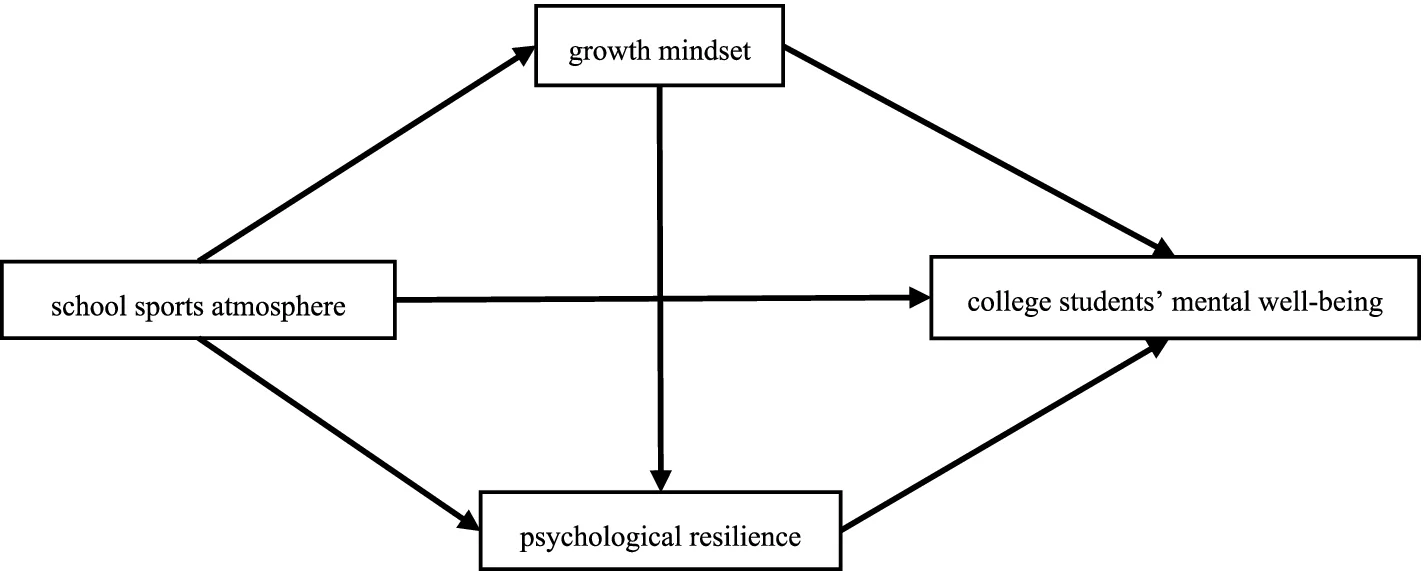
Chain mediation model of growth mindset and psychological resilience in school sports atmosphere with college students’ mental well-being.

#### Research questions and hypotheses

Accordingly, the study asks whether school sports atmosphere is related to college students’ mental well-being and whether growth mindset and psychological resilience account for independent and sequential indirect pathways in this relationship. The four hypotheses (H1-H4) specified above correspond to the direct association, the two specific indirect pathways, and the proposed sequential indirect pathway.

## Research method

### Participants

Participants were recruited from Heze Medical College using convenience sampling. Eligible participants were full-time college students enrolled in a junior college programme who voluntarily provided informed consent. Participants were 17–25 years old. In the original questionnaire, age was recorded in two grouped categories: 17–18 years (*n* = 167, 26.3%) and 19–25 years (*n* = 468, 73.7%). Of the 167 participants in the 17–18-year category, 10 were 17 years old and therefore minors at the time of data collection, whereas 157 were 18 years old; the younger age category mainly comprised first-year students. Given that research examining the relationships among school sports atmosphere, growth mindset, and psychological resilience remains relatively limited, convenience sampling was considered appropriate for this exploratory study and may provide preliminary theoretical and empirical support for future large-scale studies employing more rigorous random sampling methods. The principal investigator was responsible for the administration of the survey, which was distributed at a designated time as part of an organized activity. Before the questionnaires were administered, the researchers provided standardized instructions and emphasized the anonymous and voluntary nature of participation to minimize potential influences on participants’ responses. Students were allowed to withdraw at any point during questionnaire completion without penalty. Participants aged 18 years or older provided written informed consent themselves. For the 10 participants aged 17 years, written informed consent was obtained from a parent or legal guardian; the minor participants themselves were also informed about the study and voluntarily agreed to participate before completing the questionnaire. Participants were also explicitly informed that their responses would remain completely anonymous, that all collected data would be used solely for academic research, and that the data would be securely stored in an encrypted database. The survey was anonymous, questionnaire-based, and non-interventional. The study was conducted in accordance with the Declaration of Helsinki and was reviewed and approved by the Ethics Committee of Heze Medical College (approval document: Heze Medical College Ethics Document NO. [120]). A total of 750 questionnaires were distributed, of which 700 were returned. Based on the predefined data-quality criteria, 65 questionnaires were excluded because of an excessively short completion time, incomplete responses, or obvious patterned responding. Consequently, 635 valid questionnaires were included in the final analysis, yielding a valid response rate of 90.7%. The final sample comprised 347 male students (54.6%) and 288 female students (45.4%). Detailed characteristics of the sample are presented in Table 1. The final analytical sample size was determined by the number of returned questionnaires that satisfied the stated eligibility requirements and predefined data-quality criteria. After screening the 700 returned questionnaires, all 635 valid questionnaires were retained in the analysis; no additional subsampling was performed.

**Table 1:** Profile of the sample.

Demographics	Category	Number of people	Percentage (%)
Gender	Male	347	54.6
	Female	288	45.4
Age	17–18 years	167	26.3
	19–25 years	468	73.7
Year of study	First year	227	35.7
	Second year	214	33.7
	Third year	194	30.6
Major	Clinical medicine	147	23.1
	Nursing	198	31.2
	Medical technology	80	12.6
	Pharmacy	78	12.3
	Public services and management	123	19.4
	Other	9	1.4
Weekly exercise frequency	0 times	16	2.5
	1–2 times	332	52.3
	3–4 times	177	27.9
	5 or more times	110	17.3
Duration per exercise session	Less than 30 min	269	42.4
	30–60 min	259	40.8
	60–90 min	73	11.5
	More than 90 min	34	5.4
Participation in sports activities	Yes	450	70.9
	No	185	29.1

Of the 167 participants in the 17–18-year age category, 10 were aged 17 years (minors) and 157 were aged 18 years at the time of data collection.

### Research tool

#### School Sports Atmosphere Scale

The School Sports Atmosphere Scale was adapted with reference to the School Climate Measure developed by Zullig et al. (2010) and the adaptation approach proposed by Li et al. (2026) for the context of physical activity among Chinese college students. In the present study, the instrument was treated as a context-adapted measure based on these cited sources rather than as a newly developed scale; no independent translation-development procedure is claimed. The scale comprises 18 items across five dimensions and is rated on a five-point Likert scale ranging from 1 (“not at all true”) to 5 (“completely true”). The five dimensions are: (a) physical education teacher and instructional support (e.g., “Physical education teachers pay attention to and encourage my participation and performance in sports”); (b) connectedness through sports participation (e.g., “The school’s physical education courses or sports activities stimulate my interest in participating in exercise”); (c) fairness and order in sports activities (e.g., “Problems arising during sports activities are usually handled fairly”); (d) sports facilities and environment (e.g., “The school’s sports venues, fields, and equipment meet students’ basic needs for participating in sports activities”); and (e) peer support for sports participation (e.g., “Students around me usually support or encourage others to participate in sports activities”). In the present study, Cronbach’s *α* coefficients for the five dimensions ranged from 0.772 to 0.848, indicating good internal consistency. The results of confirmatory factor analysis showed that the second-order five-factor model had a good fit to the data: *χ*^2^/*df* = 1.721, GFI = 0.961, TLI = 0.979, IFI = 0.983, RMSEA = 0.034, and RMR = 0.039. In addition, the second-order construct of school sports atmosphere had a composite reliability of 0.888 and an average variance extracted value of 0.613, further demonstrating good convergent validity in the present sample. The retained content was required to correspond directly to one of the five sport-specific domains represented in the present instrument—physical education teacher and instructional support, connectedness through sports participation, fairness and order in sports activities, sports facilities and environment, or peer support for sports participation—so that item selection reflected the campus sports context rather than general school climate alone.

#### Growth Mindset Scale

The Growth Mindset Scale developed by Dweck was used to assess growth mindset among college students (Dweck et al., 1995). The scale primarily evaluates individuals’ beliefs regarding the malleability of intelligence or ability and includes statements reflecting both fixed and growth-oriented views. In the present study, six items were used, including three reverse-scored items (e.g., “No matter how much effort a person makes, their basic level of intelligence or ability usually cannot change substantially”) and three positively scored items (e.g., “Even when a person’s current ability is limited, they can continue to improve through learning and training”). All items were rated on a five-point Likert scale ranging from 1 (“strongly disagree”) to 5 (“strongly agree”). After the reverse-scored items were recoded, higher total scores indicated a stronger growth mindset. The six-item set was used to represent both fixed and growth-oriented beliefs about the malleability of ability, with three reverse-scored fixed-mindset items and three positively scored growth-mindset items.

The Growth Mindset Scale was administered in Chinese. The English items were translated into Chinese by a professional translator specializing in English–Chinese translation to ensure that the item meanings were conveyed accurately and were understandable to Chinese college students.

In the present study, the Growth Mindset Scale demonstrated good internal consistency, with a Cronbach’s *α* coefficient of 0.870. Confirmatory factor analysis indicated that the one-factor model provided a good fit to the data: *χ*^2^/*df* = 2.118, GFI = 0.990, TLI = 0.990, IFI = 0.994, RMSEA = 0.042, and RMR = 0.022. In addition, the scale had a composite reliability of 0.872 and an average variance extracted value of 0.533. The standardized factor loadings of the six items ranged from 0.680 to 0.840, indicating good convergent validity.

#### Connor–Davidson Resilience Scale

The Chinese version of the Connor–Davidson Resilience Scale (CD-RISC) was used to assess psychological resilience among college students. The original scale was developed by Connor and Davidson, and the reliability and validity of the Chinese version were subsequently evaluated by Yu and Zhang (2007). The Chinese CD-RISC consists of 25 items across three dimensions: tenacity (e.g., “I can remain focused and think clearly when under pressure”), strength (e.g., “I continue to make an effort until I achieve my goals”), and optimism (e.g., “I can recover quickly after experiencing difficulties”). All items were rated on a five-point Likert scale, with higher scores indicating higher levels of psychological resilience. In the present study, the Cronbach’s α coefficients for the tenacity, strength, and optimism dimensions were 0.937, 0.908, and 0.830, respectively, indicating good internal consistency. Confirmatory factor analysis showed that the second-order three-factor model provided a good fit to the data: *χ*^2^/*df* = 1.859, GFI = 0.940, TLI = 0.971, IFI = 0.974, RMSEA = 0.037, and RMR = 0.033. The second-order construct of psychological resilience had a composite reliability of 0.858 and an average variance extracted value of 0.669. The composite reliability values of the three dimensions ranged from 0.832 to 0.938, while their average variance extracted values ranged from 0.537 to 0.554, indicating good convergent validity in the present sample.

#### College students’ mental well-being

College students’ positive mental well-being was assessed using the Chinese version of the WHO-5 Well-Being Index (Fung et al., 2022). The WHO-5 is a brief self-report instrument developed from research associated with the World Health Organization and is primarily used to assess positive subjective well-being during the previous two weeks and is not a diagnostic measure of psychopathology. The scale consists of five items: (a) “I have felt cheerful and in good spirits”; (b) “I have felt calm and relaxed”; (c) “I have felt active and vigorous”; (d) “I woke up feeling fresh and rested”; and (e) “My daily life has been filled with things that interest me.” Each item was rated on a six-point scale ranging from 0 (“at no time”) to 5 (“all of the time”). The total score ranged from 0 to 25, with higher scores indicating better mental well-being. In the present study, the scale demonstrated good internal consistency, with a Cronbach’s *α* coefficient of 0.865. Confirmatory factor analysis showed that the one-factor model provided a good fit to the data: *χ*^2^/*d*f = 2.565, GFI = 0.992, TLI = 0.989, IFI = 0.994, RMSEA = 0.050, and RMR = 0.031. In addition, the scale had a composite reliability of 0.867 and an average variance extracted value of 0.568. The standardized factor loadings of the five items ranged from 0.687 to 0.849, indicating good convergent validity.

### Data processing

Data were analyzed using IBM SPSS Statistics version 28.0 and AMOS version 28.0. First, descriptive statistical analyses were conducted in SPSS 28.0 to calculate the means, standard deviations, and correlation coefficients of the key variables, including school sports atmosphere, growth mindset, psychological resilience, and college students’ mental well-being. Independent-samples t tests were used to examine gender differences in these four variables. For ease of comparison across measures, Table 2 reports mean item scores, whereas the observed score ranges reported in the Results refer to summed scale scores. Procedural steps used to reduce common method concerns included anonymous participation, standardized instructions, voluntary completion, confidentiality assurances, and use of the established response formats of the respective scales (Podsakoff et al., 2003).

**Table 2:** Descriptive statistics and correlation analysis.

Variables	M ± SD	1	2	3	4
1. School sports atmosphere	3.59 ± 0.72	1			
2. Growth mindset	3.58 ± 0.86	0.365***	1		
3. Psychological resilience	3.64 ± 0.73	0.324***	0.405***	1	
4. College students’ mental well-being	3.46 ± 1.09	0.389***	0.291***	0.466***	1

***p < 0.001.

Before testing the structural associations, a full confirmatory factor analysis was estimated with all four focal latent constructs included simultaneously. School sports atmosphere was represented as a second-order construct defined by its five first-order dimensions, psychological resilience as a second-order construct defined by tenacity, strength, and optimism, and growth mindset and mental well-being as one-factor constructs. Model fit was evaluated using *χ*^2^, degrees of freedom, *χ*^2^/*df*, NFI, IFI, TLI, CFI, RMSEA, and standardized root mean square residual (SRMR). Convergent validity was evaluated using composite reliability (CR) and average variance extracted (AVE), and discriminant validity was assessed using the Fornell–Larcker criterion and heterotrait–monotrait (HTMT) ratios, with 0.85 used as a conservative reference threshold.

A structural equation model (SEM) was established to examine the structural associations among school sports atmosphere, growth mindset, psychological resilience, and college students’ mental well-being. The model specified direct paths from school sports atmosphere to growth mindset, psychological resilience, and college students’ mental well-being; paths from growth mindset to psychological resilience and college students’ mental well-being; and a path from psychological resilience to college students’ mental well-being. Model fit was evaluated using the chi-square-to-degrees-of-freedom ratio (*χ*^2^/*df*), normed fit index (NFI), incremental fit index (IFI), Tucker–Lewis index (TLI), comparative fit index (CFI), and root mean square error of approximation (RMSEA), and standardized root mean square residual (SRMR).

The indirect effects were tested using the bootstrap method with 5,000 resamples in AMOS 28.0. Bias-corrected and percentile-based 95% confidence intervals were calculated. Three specific indirect effects were examined: the indirect effect of school sports atmosphere on college students’ mental well-being through growth mindset; the indirect effect through psychological resilience; and the sequential indirect effect through growth mindset and psychological resilience. An indirect effect was considered statistically significant when its 95% confidence interval did not include zero. All statistical tests were two-tailed, and *p* < 0.05 was considered statistically significant. Because the design was cross-sectional, directional arrows represent the theoretical specification of the model and are not interpreted as evidence of temporal precedence or causality. As a sensitivity analysis, age and gender were incorporated directly into an additional SEM as demographic covariates predicting the endogenous constructs, and the focal structural paths were compared with those in the primary model.

Alternative model assessment. Reversed sequential and parallel mediation structures were not formally tested in the present study. The hypothesized chain model was specified *a priori* on the basis of the theoretical reasoning described above. Therefore, the reported fit indices should be interpreted as evidence that the proposed model is compatible with the observed data, rather than as evidence that it is uniquely superior to all plausible alternative models. Future longitudinal research should directly compare reversed and parallel structures and examine temporal ordering.

## Result

### Assessment of common method bias

To assess potential common method bias, Harman’s single-factor test was conducted using only the 54 original measurement items (18 school sports atmosphere items, 6 growth mindset items, 25 psychological resilience items, and 5 WHO-5 items), excluding demographic variables and derived scale scores. The first unrotated factor had an eigenvalue of 15.215 and explained 28.175% of the total variance (Table 3). As a more stringent supplementary assessment, a one-factor CFA in which all 54 items were constrained to load on a single latent factor showed poor fit: *χ*^2^ = 10,219.543, *df* = 1,377, *χ*^2^/*df* = 7.422, CFI = 0.511, TLI = 0.491, IFI = 0.512, RMSEA = 0.101, and SRMR = 0.125. These findings indicate that no single common factor dominated the covariance structure. Nevertheless, because all principal constructs were assessed by self-report at the same occasion, common method variance cannot be completely ruled out (Podsakoff et al., 2003).

**Table 3:** Harman single-factor test based on the 54 original scale items.

Component	Component initial eigenvalues		
	Total	Variance %	Cumulative %
1	15.215	28.175	28.175
2	6.038	11.181	39.356
3	2.960	5.482	44.838
4	2.223	4.117	48.955
5	2.051	3.797	52.753
6	1.495	2.769	55.522
7	1.337	2.477	57.998
8	1.254	2.323	60.321

### Descriptive statistics

Table 2 presents the means, standard deviations, and Pearson correlation coefficients for school sports atmosphere, growth mindset, psychological resilience, and college students’ mental well-being. Table 4 presents the results of the independent-samples t tests examining gender differences in the four study variables. In the present sample, the observed total score ranges for school sports atmosphere, growth mindset, psychological resilience, and college students’ mental well-being were 24–85, 7–30, 28–123, and 1–25, respectively. The values reported in Table 2 are mean item scores; the observed ranges refer to summed scale scores.

**Table 4:** Gender differences in the study variables.

Variables	Gender (M ± SD)		t
	Male	Female	
School sports atmosphere	3.60 ± 0.71	3.58 ± 0.74	0.37
Growth mindset	3.59 ± 0.85	3.57 ± 0.88	0.27
Psychological resilience	3.66 ± 0.72	3.62 ± 0.74	0.76
College students’ mental well-being	3.45 ± 1.09	3.48 ± 1.09	−0.33

### Measurement model and discriminant validity

The full four-construct measurement model showed good fit to the data: *χ*^2^ = 1,934.901, *df* = 1,363, *p* < 0.001, *χ*^2^/*df* = 1.420, NFI = 0.901, IFI = 0.968, TLI = 0.967, CFI = 0.968, RMSEA = 0.026, and SRMR = 0.040. CR/AVE values were 0.888/0.613 for school sports atmosphere, 0.872/0.533 for growth mindset, 0.858/0.669 for psychological resilience, and 0.867/0.568 for mental well-being. The square roots of AVE were 0.783, 0.730, 0.818, and 0.754, respectively, all exceeding the corresponding interconstruct correlations (maximum *r* = 0.466). HTMT ratios ranged from 0.336 to 0.513, all below 0.85. Together, these results support convergent and discriminant validity among the four focal constructs.

### Structural model and indirect effect tests

The primary SEM examined the theory-specified associations among school sports atmosphere, growth mindset, psychological resilience, and college students’ mental well-being. Age and gender were not included in the primary model; instead, they were incorporated directly as demographic covariates in the sensitivity SEM reported below.

A structural equation model was constructed using AMOS 28.0. The model demonstrated a good fit to the data: *χ*^2^ = 1,934.901, *df* = 1,363, *p* < 0.001, *χ*^2^/*df* = 1.420, NFI = 0.901, IFI = 0.968, TLI = 0.967, CFI = 0.968, RMSEA = 0.026, and SRMR = 0.040. As shown in Figure 2, school sports atmosphere was significantly and positively associated with college students’ mental well-being (*β* = 0.269, *p* < 0.001), thereby supporting H1. School sports atmosphere was also positively associated with growth mindset (*β* = 0.433, *p* < 0.001) and psychological resilience (*β* = 0.245, *p* < 0.001). Growth mindset was positively associated with psychological resilience (*β* = 0.363, *p* < 0.001), while psychological resilience was positively associated with college students’ mental well-being (*β* = 0.426, *p* < 0.001). However, the direct path from growth mindset to mental well-being was not statistically significant (*β* = 0.020, *p* = 0.684). The model explained 18.7% of the variance in growth mindset (*R*^2^ = 0.187), 26.9% in psychological resilience (*R*^2^ = 0.269), and 35.9% in mental well-being (*R*^2^ = 0.359). Bootstrap analysis with 5,000 resamples was then used to evaluate the indirect effects shown in Table 5. The total effect of school sports atmosphere on mental well-being was 0.449, with a bias-corrected 95% confidence interval of [0.351, 0.537], and the direct effect was 0.269, with a 95% confidence interval of [0.193, 0.347].

**Figure 2:**
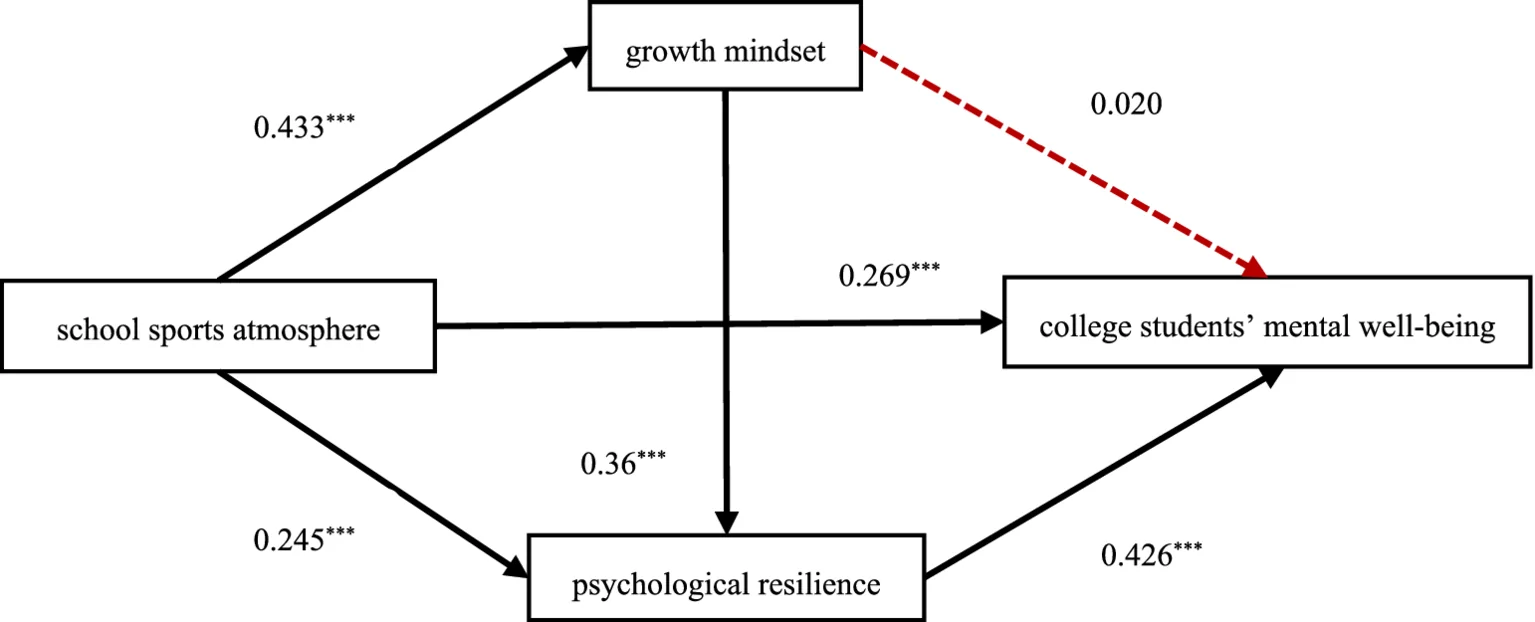
Mediating analysis path coefficient.

**Table 5:** Bootstrap tests of direct and specific indirect effects.

Model effect	Standardized effect size	Bias-Corrected		Percentile	
		95% CI		95% CI	
		Lower	Upper	Lower	Upper
Total effect	0.449	0.351	0.537	0.349	0.536
Direct effect	0.269	0.193	0.347	0.191	0.346
Indirect effect 1	0.008	−0.031	0.048	−0.032	0.047
Indirect effect 2	0.104	0.058	0.168	0.053	0.162
Indirect effect 3	0.067	0.045	0.097	0.044	0.095

Indirect effect 1: School sports atmosphere → Growth mindset → College students’ mental well-being; Indirect effect 2: School sports atmosphere → Psychological resilience → College students’ mental well-being; Indirect effect 3: School sports atmosphere → Growth mindset → Psychological resilience → College students’ mental well-being.

Among the three specific indirect pathways, the indirect effect of school sports atmosphere on mental well-being through growth mindset was 0.008, with a bias-corrected 95% confidence interval of [−0.031, 0.048]. Because the confidence interval included zero, H2 was not supported. The indirect effect through psychological resilience was 0.104, with a bias-corrected 95% confidence interval of [0.058, 0.168], supporting H3. The sequential indirect effect through growth mindset and psychological resilience was 0.067, with a bias-corrected 95% confidence interval of [0.045, 0.097], supporting H4. These indirect effects are statistical estimates from a cross-sectional model and should not be interpreted as evidence of temporal precedence or causal mediation.

A sensitivity SEM incorporated age and gender directly as demographic covariates. Model fit remained good: *χ*^2^ = 2,044.108, *df* = 1,463, *p* < 0.001, *χ*^2^/*df* = 1.397, IFI = 0.968, TLI = 0.966, CFI = 0.968, RMSEA = 0.025, and SRMR = 0.040. The focal standardized coefficients changed only minimally: school sports atmosphere → growth mindset, 0.434; school sports atmosphere → psychological resilience, 0.242; growth mindset → psychological resilience, 0.367; school sports atmosphere → mental well-being, 0.271; growth mindset → mental well-being, 0.014; and psychological resilience → mental well-being, 0.432. All originally significant focal paths remained significant, whereas the growth mindset → mental well-being path remained nonsignificant. The substantive pattern of findings was therefore robust to adjustment for age and gender.

## Discussion

### The effect of school sports atmosphere on college students’ mental well-being

The results of the present study showed that school sports atmosphere was significantly and positively associated with college students’ mental well-being, thereby supporting H1, which proposed a positive association between school sports atmosphere and college students’ mental well-being. This finding is consistent with the Social Ecological Model, which suggests that individuals’ mental health is influenced not only by personal characteristics but also by multiple levels of contextual factors, including interpersonal relationships, organizational environments, and broader social contexts (Kousoulis and Goldie, 2021). School sports atmosphere encompasses support from physical education teachers, peer interactions in sports, opportunities for sports participation, fairness of rules, and the availability of sports facilities, and therefore represents an important social-contextual resource in college students’ daily campus life (Jin and Xi, 2026; Su and Liu, 2025). A positive school sports atmosphere may provide students with greater support and more opportunities for meaningful participation in sports activities, which may in turn be associated with higher levels of psychological well-being.

This finding is also broadly consistent with previous research on the relationship between the sports environment and mental health. A positive school sports atmosphere may help reduce social anxiety among college students (Li et al., 2026). The university period is often accompanied by multiple challenges, including academic demands, interpersonal adjustment, and uncertainty about future development, while the campus serves as the primary setting for students’ learning, daily life, and social interaction (Ou et al., 2025). A positive school sports atmosphere may provide students with a form of participation that differs from academic activities, allowing them to experience positive emotions, social connectedness, and psychological relaxation through sports participation and peer interaction (Brewster and Cox, 2023). Given that the WHO-5 assesses positive mental well-being, such a supportive environment may help students maintain positive psychological states, including cheerfulness and vitality.

### The indirect role of psychological resilience

The present study examined the specific indirect effect through psychological resilience in the relationship between school sports atmosphere and college students’ mental well-being. The results showed that the indirect effect through psychological resilience was statistically significant, thereby supporting H3. This finding is consistent with the hypothesis that school sports atmosphere is indirectly associated with college students’ mental well-being through psychological resilience.

Psychological resilience refers to an individual’s capacity to maintain adaptive functioning, restore psychological balance, and continue to develop positively when confronted with stress, setbacks, or adversity. According to Conservation of Resources Theory, individuals rely on their available psychological resources when coping with stress, and psychological resilience, as a core personal resource, plays an important role in buffering the adverse effects of stress. When individuals experience resource depletion or high levels of stress, they are more likely to develop psychological imbalance, which may increase the risk of anxiety and depression (Yang et al., 2025). In contrast, a positive school sports atmosphere may help replenish psychological resources by facilitating sports participation, interpersonal interaction, and emotional release. These resources may strengthen students’ ability to adapt to academic and daily-life stressors, thereby being associated with better mental well-being (Cassaretto et al., 2024).

From the perspective of Social Support Theory, a supportive environment can enhance individuals’ sense of psychological security and belonging, thereby promoting the development of psychological resilience (Cao et al., 2024). As a distinctive social environment, school sports atmosphere not only provides college students with opportunities for emotional expression and stress relief but also strengthens social connectedness through peer interaction and collective participation (Han et al., 2025). When college students are situated in a highly supportive sports environment, they may be more likely to develop stable emotion-regulation abilities. The accumulation of these psychological resources may contribute to better overall mental well-being (Zhang and Li, 2025). Therefore, the model showed a significant indirect effect of school sports atmosphere on college students’ mental well-being through psychological resilience.

### The sequential indirect role of growth mindset and psychological resilience

The present study found a significant sequential indirect effect of school sports atmosphere on college students’ mental well-being through growth mindset and psychological resilience, thereby supporting H4.

On the one hand, a positive school sports atmosphere may provide college students with a supportive context for personal growth. Within such an environment, students are more likely to experience improvements in their abilities through sustained practice (Wu et al., 2024). On the other hand, college students with a stronger growth mindset are more likely to perceive setbacks as challenges that can be overcome through improvement. Consequently, they may be more willing to maintain their effort, adjust their strategies, and try again (Mendoza and Yan, 2025). These adaptive cognitive and behavioral responses may strengthen individuals’ capacity to persist under stressful conditions and thereby contribute to the development of psychological resilience. Psychological resilience, in turn, represents a psychological resource that is more directly related to mental well-being outcomes. When college students encounter academic pressure and uncertainty regarding their future development, higher levels of psychological resilience may help them maintain relatively stable psychological functioning and recover more rapidly from setbacks (Yang et al., 2025). Such adaptive and recovery capacities may contribute to better college students’ mental well-being.

For example, when college students experience difficulties in learning sports skills or encounter failure in physical education classes or extracurricular sports activities, a supportive school sports atmosphere may enable them, through feedback from physical education teachers and encouragement from peers, to interpret temporary failure as an outcome that can be improved through further training and strategic adjustment (Wu et al., 2024). This growth-oriented cognition may encourage students to remain engaged and respond actively to difficulties (Kroeper et al., 2022). Through repeated experiences of encountering difficulties, making adjustments, and achieving progress, students may gradually develop greater psychological resilience, which may help them maintain a more positive psychological state when facing academic demands or stressors in daily life (Ang et al., 2022; Yusefi et al., 2025).

### The indirect effect through growth mindset alone is not significant

The specific indirect effect of school sports atmosphere on college students’ mental well-being through growth mindset alone was not statistically significant. Therefore, H2 was not supported.

This finding does not suggest that growth mindset played no role in the proposed model. Rather, it indicates that growth mindset alone may not be directly associated with better mental well-being. Growth mindset primarily represents a cognitive belief in the malleability of one’s abilities. Although this belief may help individuals interpret difficulties and failures from a more positive and developmental perspective, it may not directly generate positive psychological states such as cheerfulness and relaxation (Yang et al., 2024).

When growth mindset and psychological resilience were simultaneously included in the model, growth mindset was significantly and positively associated with psychological resilience, which, in turn, was significantly and positively associated with college students’ mental well-being. This pattern suggests that growth mindset may be associated with psychological resilience by changing how individuals appraise difficulties and failures and by encouraging adjustment and proactive coping (Parada and Verlhiac, 2022). Psychological resilience may subsequently be associated with better mental well-being by strengthening individuals’ capacity to adapt to and recover from stress (Mastrokoukou et al., 2024). Growth mindset may therefore serve as a cognitive correlate of psychological resilience, whereas psychological resilience may represent an important psychological resource associated with the link between growth-oriented beliefs and mental well-being outcomes.

Relevant empirical evidence supports this more nuanced interpretation. Herdian et al. (2024) found that growth mindset was positively associated with positive mental health and psychological well-being among college students and identified perseverance of effort as an important explanatory pathway (Herdian et al., 2024). Their findings suggest that growth-oriented beliefs may relate to well-being partly through adaptive psychological resources rather than solely through a direct pathway. In the present model, psychological resilience may play a conceptually similar bridging role, although the cross-sectional design does not establish temporal mediation.

For example, college students may believe that their athletic or academic abilities can be improved through effort (Chen et al., 2026). However, when they encounter repeated failure or sustained academic pressure, growth-oriented beliefs alone may be insufficient to maintain a positive psychological state if they lack effective adjustment and coping abilities (Yang et al., 2024). Growth mindset may become significantly associated with mental well-being when growth-oriented beliefs are accompanied by persistence, strategic adjustment, and adaptive coping associated with psychological resilience.

In summary, although the stand-alone indirect effect of school sports atmosphere on college students’ mental well-being through growth mindset was not statistically significant, the sequential indirect effect through growth mindset and psychological resilience was significant. This finding suggests that growth mindset may function as a cognitive correlate within the model, whereas psychological resilience may represent an important psychological resource associated with the link between growth-oriented beliefs and positive mental well-being.

## Implications

### Optimizing school sports

Atmosphere Given the significant positive direct association between school sports atmosphere and college students’ mental well-being, universities may optimize the campus sports environment by improving sports facilities, expanding opportunities for participation, strengthening support from physical education teachers, ensuring fairness in rules, and promoting positive peer interactions (Wu et al., 2024). Physical education courses and extracurricular sports activities should provide an open, equitable, and supportive environment wherever possible, enabling college students with different levels of athletic ability and sports experience to receive positive feedback and develop meaningful participation experiences.

### Fostering growth mindset

Although the stand-alone indirect effect of school sports atmosphere on college students’ mental well-being through growth mindset was not statistically significant, the sequential indirect effect through growth mindset and psychological resilience was significant. Therefore, interventions aimed at fostering growth mindset should not be regarded as a stand-alone approach to directly improving mental well-being but should instead be integrated with the development of psychological resilience. In physical education, universities may combine process-based and value-added assessments, establish multiple learning and development goals, and help students translate growth-oriented cognitive beliefs into sustained action (Slingerland et al., 2024).

### Strengthening psychological resilience

The findings indicate a significant specific indirect effect of school sports atmosphere on college students’ mental well-being through psychological resilience. Therefore, incorporating the development of psychological resilience into university mental health promotion programmes may help strengthen students’ capacity to cope with stress and setbacks, particularly in the context of heavy academic demands and employment pressure. Specifically, universities may systematically enhance students’ psychological resilience by combining sports-based interventions, such as team sports and student club activities, with psychological skills training, including emotion regulation and stress management (Huang et al., 2024; Zhang et al., 2022).

### Pathways for promoting college students’ mental well-being

The findings suggest that a single intervention may be insufficient to comprehensively improve college students’ mental well-being. Instead, integrated interventions targeting both cognitive beliefs and psychological resources may be needed. Simultaneously fostering growth mindset and psychological resilience may help strengthen students’ positive cognitive patterns and psychological adaptability, thereby enabling them to develop more resilient coping mechanisms when confronted with stress (Mastrokoukou et al., 2024; Zhang and Li, 2025). These findings provide an important theoretical basis for universities to develop integrated intervention models that combine sports participation with psychological support.

### Future research

Future studies should employ longitudinal designs and experimental interventions to further examine the temporal and potentially causal relationships among the study variables. In addition, investigating other potential mediating or moderating variables, such as social support and self-efficacy, may contribute to a more comprehensive understanding of the mechanisms through which school sports atmosphere is associated with college students’ mental well-being.

These implications suggest that college students’ mental well-being may be systematically promoted by integrating environmental optimization with individual-level intervention strategies. This integrated approach may provide a theoretical and practical basis for the development of university mental health education and the improvement of campus physical education programmes.

## Limitations

Several limitations of the present study should be acknowledged. First, the participants were recruited from a single medical college in China. Although the sample may reflect certain characteristics of Chinese college students, it may not fully represent student populations across different cultural contexts and educational systems. Therefore, the generalizability of the findings should be interpreted with caution. Second, the present study employed a cross-sectional design. Although this design allowed the identification of associations and potential mediating pathways among the study variables, it did not permit firm conclusions regarding causality or temporal ordering. School sports atmosphere, growth mindset, and psychological resilience may change dynamically over time. Future studies should therefore employ longitudinal, cross-lagged, or experimental designs to examine temporal ordering and plausible reciprocal pathways among these variables. Third, the study relied primarily on self-reported data and may therefore have been affected by common method bias. Participants’ responses may also have been influenced by self-presentation or social desirability, potentially resulting in overestimation or underestimation of their actual experiences and psychological characteristics. This limitation may have reduced the objectivity of the data, and common method variance cannot be completely ruled out despite the additional statistical checks reported above. In addition, because the School Sports Atmosphere Scale was context-adapted from prior instruments, further validation across independent samples is warranted. Finally, although the present study examined the mediating roles of growth mindset and psychological resilience, other potentially relevant mediating or moderating variables, such as self-efficacy and emotion-regulation strategies, were not included. Future research could extend the proposed model by incorporating additional psychological and contextual factors to provide a more comprehensive and multidimensional explanation of the mechanisms underlying college students’ mental well-being.

## Conclusion

The present study found a significant positive direct association between school sports atmosphere and college students’ mental well-being. A significant specific indirect effect was observed through psychological resilience. Although the specific indirect effect through growth mindset alone was not statistically significant, growth mindset contributed to a significant sequential indirect effect through psychological resilience. These findings suggest that growth mindset may primarily serve as a cognitive correlate of psychological resilience, whereas psychological resilience may represent an important psychological resource associated with the link between growth-oriented beliefs and better mental well-being outcomes. Because the study was cross-sectional, the indirect effects should be interpreted as statistical effects within the specified model rather than as evidence of temporal or causal mediation. The findings support the continued optimization of school sports atmosphere, together with efforts to foster growth mindset and strengthen psychological resilience, as complementary approaches to promoting college students’ mental well-being. Future studies should further examine the applicability of the proposed model across different cultural contexts and populations. The model could also be extended by incorporating additional potential variables, such as social support and self-efficacy, to provide a more comprehensive understanding of the mechanisms underlying college students’ mental well-being.

## Data Availability

The raw data supporting the conclusions of this article will be made available by the authors, without undue reservation.
